# Ointments and Oleates

**Published:** 1891-03

**Authors:** 


*Ointments and Oleates.* By JOHN V. SHOEMAKER, M.D. Second Edition. Philadelphia: F. A. DAVIS. 1890.

It is rather more than ten years since Dr. Shoemaker brought out his work on Oleates, and now in this second edition he embraces the subject of ointments generally. The treatment of diseases of the skin has attained in these days to great importance, and of these diseases that of eczema, to use the expression of a very distinguished dermatologist, Professor Unna, "has long been the barometer from which we can read the exact position of dermatology of any particular period."

It is more especially in the treatment of eczema that we have associated Dr. Shoemaker's name with certain useful oleates, and chiefly those of mercury and zinc; and now we have given to us in the book before us the most recent and improved processes for their production, and also for the oleates of bismuth, copper, lead, iron, silver, tin, zinc, and a very powerful mercurous oleate, besides several others of less import; and although we cannot here go into full details about them, we can state that they each and all have their particular uses, and may often be prescribed with advantage. Then, in addition to the Oleates, rather more than two-thirds of the work are occupied by an account of ointments generally, including all those used in the British Isles, the United States, Germany, France, Austria, Italy, Spain, Mexico, and Chili. The information is very full and interesting, although not always very useful. After looking through it we were inclined to say, "Show us the Pharmacopœia of a country, and we will tell you the state of medical education there."

When we read of the arthanita ointment, with ten ingredients, of the Italian Ph., and a similar one, but very much compounded with twenty-two articles, of the Spanish Ph., we scarcely know which to be astonished at the more: the "weapon-salve" of the ancients, which had such a subtle influence that when rubbed on the weapon which had inflicted the wound it could cure the wound, whatever space intervened between the two, by a magnetic force conveyed through the air to the wound; or the compound ointments with nearly two dozen ingredients, amongst which were ox-gall, scammony, jalap, colocynth, and aloes. What a wonderful quiverful of arrows! This is polypharmacy with a vengeance!

However, we must not forget to record the many good hints and useful information given, and certain elegant preparations which are described in this part of the work. Basilicon ointment—containing varying proportions of wax, resin, and olive oil, and sometimes other things as well—seems to be a universal favourite.

Before again referring to the Oleates, we are sorry to introduce any words of censure where so much praise is due; but we must take exception to the construction of sentences here and there, as being a little slipshod and ambiguous. Thus on p. 1 is a very awkward sentence beginning with "But"; and on p. 86 we meet with a plural noun, "exudates," which we never met with before, nor is it found in a very comprehensive American lexicon which we consulted. Then the last sentence on p. 272 is very faulty, the "which appears" being not at all apparent.

However, we must conclude our remarks by expressing our indebtedness to the author for his explanations and researches about some of the more recent Oleates, and for extending the uses of the older forms, and we shall certainly return to the treatment of many skin complaints with feelings of greater confidence and security after the perusal of Dr. Shoemaker's pages.

